# Use of Cesium-131 radioactive seeds in prostate permanent implants

**DOI:** 10.4103/0971-6203.56077

**Published:** 2009

**Authors:** T. S. Kehwar

**Affiliations:** Member Editorial Board, JMP, Department of Radiation Oncology, University of Pittsburgh Cancer Institute, UPMC Cancer Centers, Pittsburgh, PA 15232, USA. E-mail: drkehwar@gmail.com

Permanent prostate seed implantation is a minimally invasive approach to implant low activity radioactive sources to the prostate gland with the guidance of transperineal ultrasound technique. Historically, radium was used therapeutically within a decade of its discovery for intracavitary prostate brachytherapy.[1] Naturally occurring radioisotopes are not ideal sources for permanent seed implantation. One of the first man-made radioisotopes was Gold - 198 (^198^Au)[2] used for prostate permanent seed implantation. In the modern era of prostate implant brachytherapy, two radioisotopes - iodine-125 (^125^I) and palladium-103 (^103^Pd) have extensively been used for the purpose.[3]

Recently, a new cesium -131 (^131^Cs) radioactive seed has been introduced (Iso Ray, Richmond, WA) in the clinical practice for permanent seed implants of early prostate cancer.[4] ^131^Cs is a 4.5 mm × 0.8 mm seed, titanium - encased ceramic with gold wire. Since being introduced in 2004, about 3000 prostate implants using ^131^Cs seeds have been performed.[5]

The introduction of ^131^Cs seed, for prostate permanent implants, is not without controversy and some researchers oppose its use stating that there is not enough data on dosimetric and clinical use of this source. However, our experience at UPMC, Pittsburgh, in prostate permanent seed implants using ^131^Cs source is encouraging.[6]

The question arises - what treatment modality is better for a particular patient and if it is decided that patient has to go for permanent seed implant then which radioisotope should be used. This can be decided following a discussion between the physicist, physician and patient based on the status of the disease and general condition of the patient. The choice of radioactive seed to be used is purely based on the physician's feedback. This includes the characteristics of the disease, possible outcome and complications with the use of a particular isotope. The intention to write this editorial is to outline our present level of knowledge about ^131^Cs seed implants on the basis of radiobiological, radiological and clinical aspects.

Before discussing ^131^Cs implants let us first briefly describe the characteristics of prostate implants.

1. For an effective permanent prostate seed implant, the radioactive source should have short half life and low photon energy. The half lives and average photon energies are 60 days and 27 keV for ^125^I, 17 days and 23 keV for ^103^Pd and 9.7 days and 29 keV for ^131^Cs, respectively. The ^131^Cs isotope was first evaluated by Henschke and Lawrance[4] which is an x-ray emitter with its most prominent energy peaks in the range of 29 - 34 keV. ^131^Cs meets the criteria with the half life considerably shorter than the half lives of ^125^I and ^103^Pd sources. Thereby, ^131^Cs offers higher initial dose rate and delivers total dose in a shorter period of time.
  Since ^131^Cs is a new source therefore no standard has been established at NIST (or at any other competent / regulatory body). It is important that an extensive study must be done to determine its dosimetric and radiologic parameters. A number of studies had been done to do so in accordance of AAPM TG 43 report,[7] which includes both empirical and Monte Carlo techniques.
2. The distribution of ^125^I and ^103^Pd seeds is being done using following approaches:
  - Uniform: Seeds are placed on a 1.0 cm × 1.0 cm × 1.0 cm grid throughout the prostate volume.
  - Modified Uniform: Seeds are distributed as in uniform except that certain central positions are avoided to get urethral dose less than 150 % of the prescribed dose.
  - Peripheral: Seeds are placed around the inner periphery of the prostate, and
  - Modified Peripheral: Seeds are placed around the inner periphery of the prostate along with some seeds placed centrally to get homogeneous dose distribution.
3. Dose rate effect: In prostate permanent seed implants, the total dose to the prostate is prescribed taking into account the source half life, i.e. dose rate effect, which is an important factor in the prescription of the prostate implant dose. The generally prescribed dose for prostate is 145 Gy for ^125^I, 124 Gy for ^103^Pd and 115 Gy for ^131^Cs, for monotherapy treatments corresponding to the biologically effective doses of 111 Gy, 115 Gy and 112 Gy respectively.[8] Approximately, 15 and 20% dose rate corrections were applied to the prescribed dose of ^125^I to get prescribed doses for ^103^Pd and ^131^Cs seeds, respectively. For boost therapy following external beam radiation therapy (EBRT), it is 100 Gy, 82 Gy and 80 - 85 Gy for ^125^I, ^103^Pd and ^131^Cs seeds, respectively.
4. Effect of Prostatic edema: Prostatic edema develops during source implantation procedure. In ^131^Cs implants the effect of edema is very much pronounced because most of the dose is delivered before prostate reaches its pre implant size.[9]

Since average photon energy of ^131^Cs source is slightly higher than that of ^125^I and ^103^Pd sources, in UPMC, the modified peripheral approach is used in the seed implantation within prostate gland. Central seeds are implanted no closer than 0.5 cm to the urethra to keep urethral dose less than 150% of the prescribed dose.

## Radiobiological Facts

Use of different radioisotopes in permanent prostate seed implantation poses radiobiological challenges. During the time period taken to deliver the prescribed dose, prostate cancer cells, may have significant tumor cell repopulation and late reacting tissues in the vicinity of the prostate will continue to be damaged. The probability of sterilizing remaining tumor cells will also decrease as dose rate falls and may result in a considerable amount of dose being wasted. Armpilia et al.[10] have analyzed radiobiological parameters and found that rapidly growing tumors require prescribed dose to be delivered in the shortest period of time, which can be done only by a radionuclide with shortest half-life. Even slow growing tumors, such as prostate adenocarcinomas, can be satisfactorily treated with a radionuclide possessing half life substantially less than that of ^125^I. The value of α/β for prostate is comparable with or lower than that of the late reacting normal tissues[10] which mitigates against the use of long lived radioisotopes for permanent implants.

Armpilia and his colleagues[10] calculated optimum half lives of radionuclides ranging from zero to five days for fast repopulating tumors to approximately 14 - 50 days for slow growing tumors. This suggests that for prostate implantation, for a wide variety of tumors, shorter lived radioisotopes are more capable of achieving sensible clinical results than longer lived isotopes. In comparison to the above described results, the ^131^Cs source would be even better than both ^125^I and ^103^Pd sources.

Kehwar *et al*.[9] studied that with an account of prostatic edema, at the time of implant procedure, ^131^Cs seeds deliver about 85% of the planned dose in four weeks of implant when edema effect is maximum. During this period of treatment, the dose rate is higher and sufficient enough to encounter fast proliferative cells before initiation of excess proliferation.

## Clinical Facts

Since first prostate implant in 2004 with ^131^Cs, a number of clinical trials were conducted to evaluate the outcome of monotherapy and boost therapy for external beam radiotherapy. Although proper characterization of dosimetric parameters of radioactive sources,[4,8] dose distribution within target volume and surrounding normal tissues and related radiobiological analysis are important factor in deciding the use of a radionuclide in prostate implants, the only true test for a radiation oncologist is to - how good it works clinically.

A group of radiation oncologists and physicist[11] have performed more than 1200 prostate implants using ^131^Cs seeds and promulgated a set of recommendations for prescribed dose, dose constant, radiation safety, post implant imaging etc. Results indicate that ^131^Cs, with its short half life, has somewhat more intense urinary and rectal complication but seems to resolve more quickly than that with ^125^I and ^103^Pd. ^131^Cs has a higher average photon energy that contributes better uniform dose distribution within target volume but can push an unwanted dose to rectum and bladder. With a maximum follow-up of just over three years and median follow-up of 23 months, the drop in prostate-specific antigen levels from cesium appears equivalent to that from the other isotopes.

When ^103^Pd was introduced in the early 1990 as an alternative to ^125^I for prostate implants, similar questions had been raised. Almost similar radiobiological arguments were put forward then, as now, for ^131^Cs. The value of α/β ratio was used higher during that period of time than today. We have to answer these questions and put forward radiobiological arguments which employ the need for an even more aggressive, shorter half life isotope.[10] In reality, radiation oncologists are more cautious than any other medical communities in adopting new treatments as “radiation causes damage in healthy tissues as well”. It is known that ^131^Cs has a higher energy than either ^125^I or ^103^Pd and the dose is delivered more quickly than by ^125^I or ^103^Pd isotopes. Hence practicing radiation oncologists must pay attention to the design of seed positioning with an account of prostatic edema, dose distribution, dose rate effect and of course to the results of clinical trials and panel recommendations. The use of ^131^Cs seeds in permanent prostate implantation can be done adequately by any radiation oncologist who has performed prostate implants with either ^125^I or ^103^Pd or both. There is no reason to fear misapplication of the source which even can just as well happen with iodine or palladium. Higher average energy of ^131^Cs may push more doses to rectum, but this also makes more homogeneous dose distribution within prostate gland and offers better coverage. As discussed earlier, the use of ^131^Cs seeds would offer better clinical results in fast repopulating tumor cells. An increased dose rate may make patients more susceptible to early complications, but it also makes them less susceptible to late ones.
